# Band-by-band spectral radiative kernels based on the ERA5 reanalysis

**DOI:** 10.1038/s41597-024-03080-y

**Published:** 2024-02-24

**Authors:** Han Huang, Yi Huang, Qiang Wei, Yongyun Hu

**Affiliations:** 1 Department of Atmospheric and Oceanic Sciences, McGill University, Montreal, Canada; 2 State Key Laboratory of Tibetan Plateau Earth System, Environment and Resources (TPESER), Institute of Tibetan Plateau Research, Chinese Academy of Sciences, Beijing, China; 3 Department of Atmospheric and Oceanic Sciences, Peking University, Beijing, China

**Keywords:** Atmospheric science, Climate change

## Abstract

Radiative kernel is a widely adopted method for diagnosing radiation variability and climate feedback. However, most of the existing radiative kernels are broadband flux kernels and lack the spectral information. Motivated by the growing interest in the spectral changes of the Earth radiation budget, we generate a new set of band-by-band radiative kernels based on the fifth generation European Center for Medium-Range Weather Forecasts (ERA5) reanalysis, which can be used for analyzing the spectrally decomposed changes in the top of atmosphere, surface and atmospheric radiation. The radiative sensitivity quantified by the ERA5 band-by-band kernel is compared to another spectral kernel and validated in a spectral radiation closure test. The use and benefits of the new ERA5 kernels are then demonstrated in an analysis of spectral feedbacks of an ensemble of global climate models (GCMs).

## Background & Summary

Radiative kernels have become an essential tool for diagnosing the Earth’s radiation budget. Given its efficiency in quantifying radiative feedbacks, multiple sets of radiative kernels based on different atmospheric datasets have been developed and widely used for diagnosing the climate feedbacks in global climate models (GCMs) and observations^1–12^. However, most of these kernels only measure the sensitivity of broadband fluxes, except for several developments^6,13,14^ that made use of longwave spectral kernels.

On the other hand, the benefits of spectral kernels are obvious. A number of studies have demonstrated how climate changes can be better detected by radiation spectral changes^15–22^ and can be attributed based on the distinctive spectral signatures of radiative forcing and feedback^19,23,24^. A growing interest especially worth noting is that in the far-infrared (FIR) spectrum, which is crucial for the Earth energy budget^25–29^ and has motivated ongoing development of several satellites^26–28^. Spectrally decomposed kernels would facilitate the dissection of Earth radiation budget, including that in the FIR, and help identify the major geophysical variables accounting for the radiation variability.

In this paper, building on the recent development of a set of broadband kernels^12^, we generate a new set of spectrally decomposed radiative kernels for the top of atmosphere (TOA), surface and atmospheric radiation, respectively. We validate the use of them in explaining simulated spectral radiation changes and then demonstrate their use in diagnosing the radiative feedbacks in an ensemble of GCMs in comparison with another spectral kernel dataset. As the new kernels generated here measure radiative sensitivities in individual spectral bands (spectral intervals in the order of 100 cm^−1^), we refer to them as “spectral” kernels for simplicity. Note, however, their resolution should be distinguished from the spectral kernels at 10 cm^−1^ or finer resolutions, which have been developed elsewhere^6,13,30^.

## Methods

The spectral kernels developed here are based on the same perturbation experiments used by Huang and Huang^12^ to produce the broadband flux kernels based on the fifth generation European Center for Medium-Range Weather Forecasts (ERA5) reanalysis^31^. The instantaneous profiles of surface and air temperature, atmospheric water vapor, cloud cover and cloud water path are from the ERA5 reanalysis. The cloud effective radii are from the 3-hourly synoptic TOA and surface fluxes and cloud product of the Clouds and Earth’s Radiant Energy System (CERES)^32^. Five more layers of the U.S. standard profile are patched above 1hPa for LW calculation, to ensure the accuracy of radiative kernels in the upper atmosphere^11^. Random cloud overlapping scheme is used for all-sky calculation as different cloud overlapping schemes show similar simulation results. Effective surface emissivity inferred from the ERA5 broadband surface radiative fluxes is used in the calculation. More details can be found in Huang and Huang^12^. The kernels measure the radiative sensitivity to surface temperature (*T*_*s*_), air temperature (*T*_*a*_), and water vapor (*WV LW*) in longwave radiation and to surface albedo (*ALB*) and water vapor (*WV SW*) in shortwave. They are generated using the Rapid Radiative Transfer Model (RRTMG)^33^ and instantaneous ERA5 profiles with a horizontal resolution of 2.5 degree × 2.5 degree and a vertical resolution of 37 pressure levels from 1000hPa to 1hPa. A five-year (2011–2015) simulation is conducted to generate the multi-year monthly mean ERA5 spectral kernels.

Different from the broadband kernels, the spectral kernels decompose the radiative sensitivity to 16 LW spectral bands and 14 SW spectral bands, taking advantage of the band configuration of the RRTMG (see Table 1). For example, the produced air temperature spectral kernel is a 5-dimentional (5-D) array of month|12 × level|37 × latitude|73 × longitude|144 × LW_band|16 and the surface albedo spectral kernel is a 4-D array of month|12 × latitude|73 × longitude|144 × SW_band|14. For a more detailed documentation of the kernel computation procedure, we refer interested readers to Huang and Huang^12^. Note that all radiative fluxes and kernel values are defined downward positive. For example, a positive LW kernel value means less outgoing longwave radiation (OLR) at the TOA, more downwelling radiative flux at the surface, or more radiative flux convergence in the atmosphere.

**Table 1:** LW and SW bands in spectral kernels.

LW band	Wavenumber (cm^−1^)	SW band	Wavenumber (cm^−1^)
1	10–350	1	820–2600
2	350–500	2	2600–3250
3	500–630	3	3250–4000
4	630–700	4	4000–4650
5	700–820	5	4650–5150
6	820–980	6	5150–6150
7	980–1080	7	6150–7700
8	1080–1180	8	7700–8050
9	1180–1390	9	8050–12850
10	1390–1480	10	12850–16000
11	1480–1800	11	16000–22650
12	1800–2080	12	22650–29000
13	2080–2250	13	29000–38000
14	2250–2380	14	38000–50000
15	2380–2600		
16	2600–3250		

## Data Records

To not repeat the thorough documentation of the ERA5 kernels in Huang and Huang^12^, we exemplify the spectral kernels by showing their global mean values in the following. In a summary NetCDF data file stored in Mendeley repository^34^, we record the global mean values, as well as other summaries of the kernels including the zonal mean values and vertically integrated values, for interested reader to have an overview of them. The ERA5 spectral kernels contain the surface temperature (*T*_*s*_), air temperature (*T*_*a*_), water vapor LW (*WV LW*), surface albedo (*ALB*) and water vapor SW (*WV SW*) kernels, with the resolutions described in Methods part. For the air temperature and water vapor kernels (*WV LW* and *WV SW*), two versions of these kernels are provided, with one normalized by layer thickness and another representing the specific layer.

Another spectral kernel generated by Huang *et al*.^6^ (referred to as H14 kernel hereafter), which is interpolated to the same resolution of ERA5 kernel for comparison is also provided in the repository. The feedback results as illustrated in Figs. 4, 5 are also included.

Note that all kernels shown here are normalized with band width if not otherwise mentioned and represent radiative sensitivity in unit wavenumber interval. For example, the spectral surface temperature kernel is in units of W (m^2^ K cm^−1^)^−1^.

**Fig. 1:**
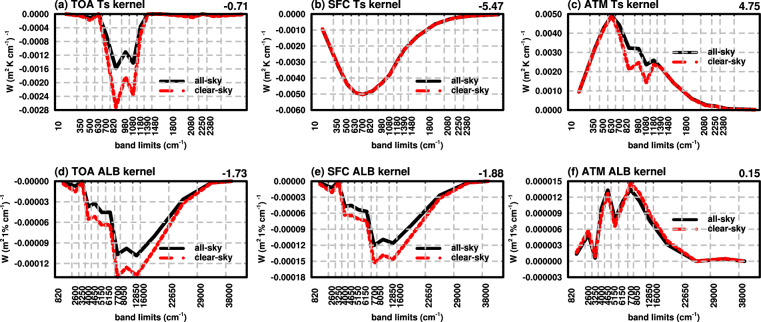
Surface temperature spectral kernels for (**a**) TOA, (**b**) surface and (**c**) atmosphere radiative fluxes, units: W (m^2^ K cm^−1^)^−1^. (**d,e,f**) Similar to (**a,b,c**), but for surface albedo kernels, units: W (m^2^ 1% cm^−1^)^−1^. Black solid lines are results in all-sky and red dashed lines are results in clear-sky. The numbers on the right corner are the spectrally integrated (i.e., broadband) global mean sensitivity values in all-sky.

We first present the spectral distribution of radiative sensitivities. Figure 1 shows the global mean ERA5 spectral kernels of single-level variables, i.e., surface temperature and surface albedo. For the TOA flux kernel of surface temperature (Fig. 1a), the main contributions are from the window region (820–1080 cm^−1^) due to the weak atmospheric absorption and higher transmittance in this spectral band. The sensitivity in all-sky is weaker than that in clear-sky due to the masking effect of cloud. For the surface flux kernel of surface temperature (Fig. 1b), as its change is mainly affected by the upward emission from the surface, the spectral distributions in clear-sky and all-sky are almost identical, both of which primarily follow the Planck function. For the kernel of atmospheric radiation defined by differencing TOA and surface radiation flux (Fig. 1c), the difference between the all-sky and clear-sky kernels again exists in the window region, as the strong absorption in other bands makes the absorption of surface emission by the atmosphere insensitive to cloud; the higher value in all-sky is because clouds enhance the absorption of surface emission.

For the surface albedo kernels, the major contributions are from 7700 cm^−1^ to 22650 cm^−1^ (from 440 nm to 1300 nm) which mainly lie in the visible and near-infrared bands. The sensitivities of both TOA (Fig. 1d) and surface (Fig. 1e) fluxes in all-sky are weaker than those in clear-sky as the SW reflection by clouds reduces the radiative flux reaching the surface and thus the sensitivity to surface albedo. The atmospheric radiation sensitivity to surface albedo is positive (Fig. 1f) as the increase in surface albedo enhances the SW reflection between the atmosphere and surface and meanwhile the SW absorption in the atmosphere.

**Fig. 2:**
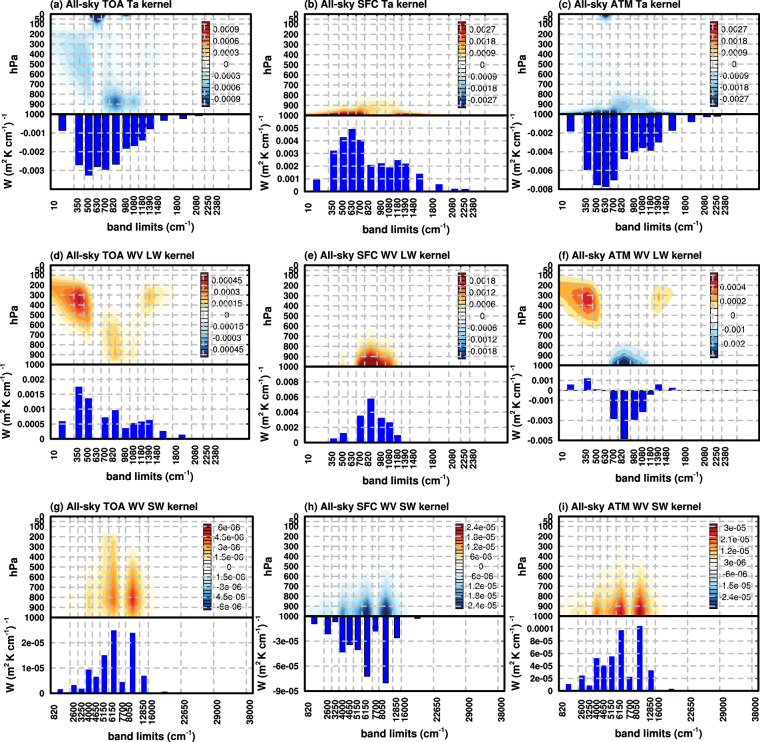
All-sky spectral air temperature kernels of (**a**) TOA, (**b**) surface and (**c**) atmospheric radiative fluxes. (**d,e,f**) and (**g,h,i**) Similar to (**a,b,c**), but for water vapor LW kernel and water vapor SW kernel, respectively. Contour plots are vertically decomposed spectral kernels, units: W (m^2^ K cm^−1^ 100hPa)^−1^. Bar plots are vertically integrated spectral kernels, units: W (m^2^ K cm^−1^)^−1^.

Figure 2 shows the global mean spectral kernels of air temperature, water vapor LW and water vapor SW in all-sky. Clear-sky kernels have similar patterns and are shown in the Supplementary Figure S1.

For the air temperature kernels of the TOA flux (Fig. 2a), the radiative fluxes in far-infrared bands (10–630 cm^−1^) are mostly sensitive to the perturbation in the mid-to-high troposphere due to strong absorption of water vapor in this spectral region. In CO_2_ absorption bands (700–820 cm^−1^), the signal is only visible in the stratosphere as the strong masking effect of CO_2_ absorption in this band. In the window region (820–1080 cm^−1^), due to relatively higher transmittance in the atmosphere, the radiative sensitivity maximizes in the lower troposphere. In the ozone absorption band (1080–1180 cm^−1^), similar to CO_2_ absorption band, the OLR change is mostly sensitive to the air temperature change in the stratosphere. In the 1390–3250 cm^−1^ region, the sensitivity is weak due to the relatively lower energy of Planck function at Earth’s emitting temperature in this band. Unlike the TOA kernels, the air temperature kernel of the surface flux (Fig. 2b) is concentrated in the lower atmosphere, with stronger contributions from water vapor and CO_2_ absorption bands (e.g., 10–630 cm^−1^ and 630–800 cm^−1^) than the window region. For the kernels of atmospheric radiation (Fig. 2c), it is dominated by emitters near its boundaries, including the water vapor and CO_2_ molecules in the lower troposphere (near the lower boundary) and the CO_2_ and O_3_ in stratosphere (near the top boundary).

For the water vapor LW kernels, the TOA kernels (Fig. 2d) show strong sensitivity in the upper troposphere in water vapor absorption bands, especially its rotational band in the FIR (10–630 cm^−1^), and the sensitivity in the window region is mainly to the mid-to-low troposphere. For water vapor LW kernels of surface flux (Fig. 2e), the downwelling radiative flux at the surface is almost only sensitive to the water vapor perturbations in the window regions. This is because the absorption in water vapor absorbing bands is largely saturated in the lowest layers, which renders little radiative sensitivity to water vapor perturbations. For the kernels of atmospheric radiation (Fig. 2e), the sign varies with spectral bands. In water vapor absorption bands, the increase of water vapor leads to more energy being trapped in the atmosphere and warms the atmosphere, while in the window region more water vapor results in more emission from the atmosphere and thus an energy loss in the atmosphere. From the vertically integrated results (bar plot in Fig. 2f), this cooling effect in the window region dominates the warming effect in the water vapor absorption bands.

From the water vapor SW kernels, the increase in water vapor concentration reduces the reflected SW at the TOA and the SW reaching the surface and enhances the absorption of SW in the atmosphere. Hence, the TOA (Fig. 2g) and atmospheric kernels (Fig. 2i) are positive and the surface kernel (Fig. 2h) is negative. There are three most sensitive spectral regions (4000–4650 cm^−1^, 6150–7700 cm^−1^ and 8050–12850 cm^−1^), mainly in the infrared region. The TOA kernels peak at the lower troposphere (around 900hPa) while surface and atmosphere kernels peak at the bottom layer.

The air temperature and water vapor LW kernels, although spectrally decomposed only to 16 bands, resemble those generated with high spectral resolution models e.g., Huang, *et al*.^35^, evidencing that the ERA5 spectral kernels generated here capture the major spectral features of the radiative sensitivity. Moreover, the TOA kernels resemble the vertically decomposed atmospheric contribution to OLR as shown in Huang and Huang^29^, corroborating the fact that the OLR in different bands mostly originates from different vertical layers.

## Technical Validation

Several validation tests have been conducted for this new kernel dataset, including a linearity additivity test, in which the vertical sum of radiative kernels is shown to reproduce the radiative flux change caused by a vertically uniform perturbation, consistent with what was reported by Huang and Huang^12^.

To further validate the ERA5 spectral kernels, we compare them with those generated by Huang, *et al*.^6^ (H14 kernel), which are based on the atmosphere of a GCM of the Geophysical Fluid Dynamics Laboratory (GFDL). The comparison is limited to TOA LW kernels as the H14 spectral kernels are only available for TOA LW. The H14 kernels are generated in high spectral resolution but integrated to match the 16 bands in LW and 14 bands in SW, as in the ERA5 spectral kernels.

**Fig. 3:**
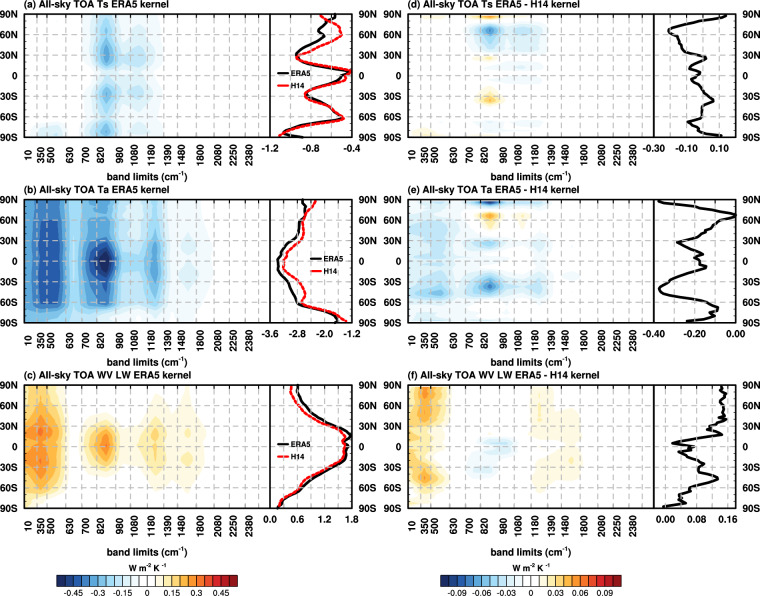
A comparison of ERA5 and H14 all-sky LW spectral kernels. In color-contours are the zonal mean band-by-band values of ERA5 kernels of (**a**) surface temperature and vertically integrated kernel values of (**b**) air temperature and (**c**) water vapor, units: W m^−2^ K^−1^. Line plots in panels (**a**–**c**) are the broadband zonal mean kernel values of the ERA5 (black) and H14 (red) kernels. Panels (**d**–**f**) similar to panels (**a**–**c**), but the difference between ERA5 and H14 kernels (ERA5 minus H14).

Panels a-c of Fig. 3 show the zonal mean band-by-band ERA5 spectral kernels (units: W m^−2^ K^−1^), which show spectral sensitivities across different latitudes. Some features shown by the global mean kernels in Figs. 1, 2 are found to be persistent at all latitudes. However, there are also latitudinal differences.

Panels d-f of Fig. 3 show the differences between ERA5 and H14 kernels. In general, the two datasets show good agreements, evidenced by the generally much smaller discrepancies compared to the kernel values in terms of their magnitudes. Some relatively larger discrepancies are noticed, such as the surface temperature kernel in the high latitude of Northern Hemisphere (Fig. 3d), which is mainly from the window region. For air temperature and water vapor LW kernels, the differences are from both water vapor absorption bands and window region (Fig. 3e,f). Nevertheless, these quantitative differences lead to little discrepancies in the global mean feedback values quantified from the two sets of kernels (see Fig. 5).

## Usage Notes

To demonstrate the use of the spectral kernels, we apply them in two climate change problems. First, we conduct a radiation closure test by applying the ERA5 spectral kernels to diagnosing the feedback in El Niño and Southern Oscillation (ENSO) and compare the kernel-diagnosed results to RRTMG-computed (*truth*) spectrally decomposed radiation changes. Then, we apply the ERA5 spectral kernels to quantify the spectrally decomposed radiative feedbacks in an ensemble of GCMs in a quadrupling CO_2_ experiment, in comparison with the longwave spectral kernels of Huang *et al*.^6^.

### ENSO

To acquire a reference (*truth*) of radiation changes for validating the kernel diagnosis, we simulate the TOA radiation changes between 2015 (a strong El Niño year) and 2011 (a strong La Nina year) using the RRTMG and 4-times daily instantaneous ERA5 profiles. We then compare the spectrally decomposed annual mean TOA radiation changes in the 30 bands (see Table 1) simulated from RRTMG with those diagnosed from the ERA5 spectral radiative kernels.

As the kernels in question here are the kernels of non-cloud variables, we focus on the clear-sky radiation change for validating the radiation closure, although the cloud feedback can be obtained by using the adjusted cloud radiative effect method (Shell, *et al*.^2^). In the clear-sky, each non-cloud spectral radiative feedback $\Delta {R}_{i,X}^{o}$ is calculated as:

1 $$\Delta {R}_{i,X}^{o}={K}_{i,X}^{o}\cdot \Delta X$$

which is in the units of W m^−2^. Here, the superscript *o* indicates results in clear-sky and the subscript *i* denotes the *i*-th spectral band (among 16 LW bands and 14 SW bands as listed in Table 1). ${K}_{i,X}^{o}$ is the clear sky spectral kernel of variable *X* in the i-th spectral band. ∆*X* is the anomaly of variable *X* (surface temperature, air temperature, water vapor or surface albedo) measured by the difference between the two years (2015 minus 2011). Note for the water vapor feedback, a logarithmic scaling is used following most feedback analyses. As explained by Huang and Huang^12^, we use the vertical layer-specified kernels rather than layer thickness-normalized kernels in computing the air temperature and water vapor feedbacks. Note that here we report the band integrated spectral feedbacks (in the units of W m^−2^, as opposed to W m^−2^ (cm^−1^)^−1^). Hence the corresponding broadband radiation changes can be calculated as the sum of changes in each individual band.

The residual term in each band $\Delta {R}_{i,res}^{0}$ is calculated as:

2 $$\Delta {R}_{i,res}^{0}=\Delta {R}_{i}^{0}-{\sum }_{X}\Delta {R}_{i,X}^{o}$$

where $\Delta {R}_{i}^{0}$ is the spectrally decomposed radiation change in the *i*-th spectral band simulated by RRTMG, ${\sum }_{x}\Delta {R}_{i,x}^{0}$ is the sum of all non-cloud feedbacks diagnosed by the spectral kernels. The broadband residual $\Delta {R}_{res}^{0}$ can be obtained by summing all the band residuals.

**Fig. 4:**
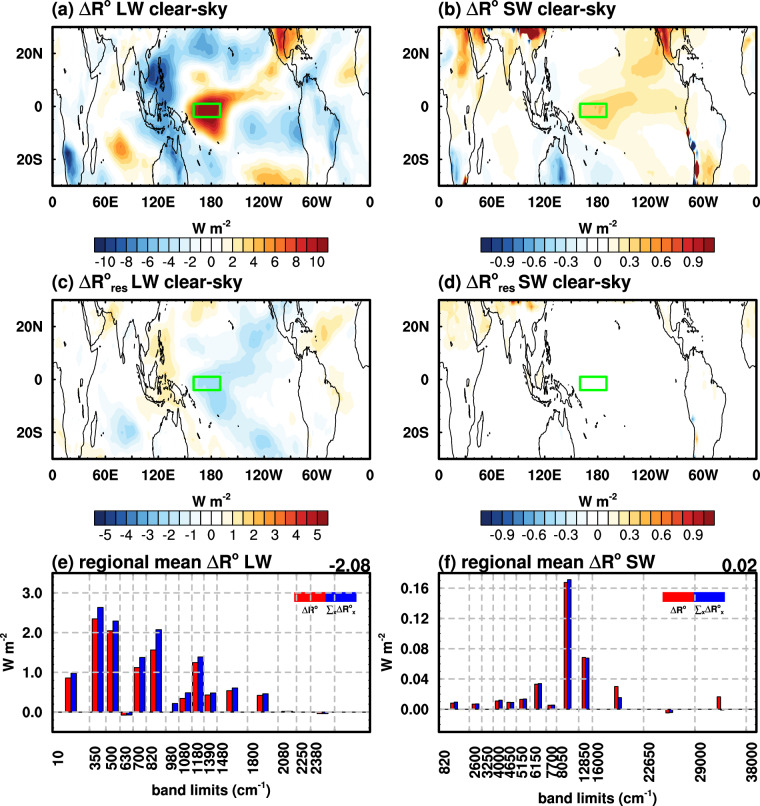
Clear-sky (**a,b**) broadband radiation change simulated by RRTMG for LW and SW, (**c,d**) residual terms quantified by ERA5 spectral kernels for LW and SW, (**e,f**) regional mean (as marked by the green rectangle) spectral radiation change in each band diagnosed by ERA5 spectral radiative kernels during ENSO. The numbers on the right corner in (**e,f**) are the regional mean residual terms.

ENSO is characterized by a movement of convective systems from the Warm Pool region to the Central Pacific region. As shown by Fig. 4a,b, there is positive LW anomalies in the Central Pacific and negative anomalies in the Warm Pool region in the El Nino year (2015) compared to the La Nina year (2011). Figure 4c,d show the broadband residual terms in LW and SW quantified by the multi-year mean ERA5 spectral kernels; similar results using H14 spectral kernels and ERA5 results for the surface are shown in Supplementary Figures S2, S3, respectively, which demonstrate similar, good performance of the kernel method.

In the region of the most prominent local feedback (longitudes 160E–190E, latitudes 1N-4S, as marked by the green rectangle on Fig. 4a), we take a close examination of the spectral feedbacks in Fig. 4c,d. In this region, due to local surface warming (a maximum temperature anomaly of 2.5 K and an annual mean anomaly of 1.8 K between 2015 and 2011), a total radiation change (∆*R*^*o*^) of 10.82 W m^−2^ and 0.36 W m^−2^ is measured in longwave and shortwave, respectively, exhibiting a super-greenhouse behavior^16^. The multi-year mean ERA5 spectral kernels, can well reproduce the total radiation changes simulated by RRTMG in both longwave and shortwave.

In the shortwave, kernel results also generally well reproduce the RRTMG-simulated total radiation changes (Fig. 4f). An interesting, relatively larger bias there is in the band of 16000–38000 cm^−1^. This is due to the radiative effect of ozone. When simulating the radiation changes between the two years (2015 and 2011), different ozone concentrations appropriate to each year (as given by the ERA5 dataset) are used in the RRTMG simulation. This suggests a full closure of the radiation budget may be better achieved with ozone kernels, which warrants future development.

In summary, by validating the kernel-diagnosed spectral radiation flux changes against RRTMG simulations, our results demonstrate that the ERA5 spectral kernels well capture the major spectral features of longwave and shortwave radiation changes, which evidences the validity of the spectral kernels.

### Quadrupling CO_2_

In this part, we use the abrupt4xCO_2_^36^ and piClim-4xCO_2_^37^ simulations from 12 CMIP6 models (see Table 2) to demonstrate the application of ERA5 spectral kernels to analyzing the climate feedbacks in GCMs. The abrupt4xCO_2_ experiment is simulated with an instantaneous quadrupling of CO_2_ concentration of year 1850 and the piClim-4xCO_2_ simulation is modeled with SST and sea ice concentrations fixed at the climatology of pre-industrial control experiment and CO_2_ concentration quadrupled. To quantify the radiative feedbacks, the last 20-year data in each experiment is used and interpolated to the same horizontal and vertical grids of the spectral kernels.

**Table 2:** CMIP6 models used in this study.

Models	Horizontal resolution (lat*lon)	Vertical levels	Reference
ACCESS-ESM1-5	1.25*1.875	38 levels to 40 km	Ziehn, *et al*.^40^
CanESM5	2.8*2.8	49 levels to 1hPa	Swart, *et al*.^41^
CESM2	0.9*1.25	32 levels to 2.26hPa	Danabasoglu, *et al*.^42^
CNRM-CM6-1	1.4*1.4	91 levels to 0.01hPa	Voldoire, *et al*.^43^
EC-Earth3	0.7*0.7	91 levels to 90 km	Döscher, *et al*.^44^
GISS-E2-1-G	2*2.5	40 levels to 0.1hPa	Kelley, *et al*.^45^
HadGEM3-GC31-LL	1.25*1.875	85 levels to 85 km	Williams, *et al*.^46^
IPSL-CM6A-LR	1.3*2.5	79 levels to 80 km	Boucher, *et al*.^47^
MIROC6	1.4*1.4	81 levels to 0.004hPa	Tatebe, *et al*.^48^
MPI-ESM1-2-LR	1.875*1.875	47 levels to 0.01hPa	Mauritsen, *et al*.^49^
MRI-ESM2-0	1.125*1.125	80 levels to 0.01hPa	Yukimoto, *et al*.^50^
UKESM1-0-LL	1.25*1.875	85 levels to 85 km	Sellar, *et al*.^51^

Following Eq. (1) and normalizing the spectral radiative feedback ∆*R*_*X*_ with the global mean surface temperature change ∆*T*_*s*_ in each model, the spectral feedback parameter *λ*_*X*_ is obtained as:

3 $${\lambda }_{i,x}=\frac{\Delta {R}_{i,x}}{\Delta {T}_{s}}$$

in units of W m^−2^ K^−1^. For longwave feedbacks, *λ*_*i,X*_ has 16 spectral bands and for shortwave feedbacks, 14 spectral bands. For readers who are interested in the spectral feedback intensity parameter in units of W (m^2^ K cm^−1^)^−1^, results are shown in Supplementary Figure S4.

As the spectrally decomposed total radiation changes are not available from the GCM simulations, we focus on the all-sky non-cloud feedbacks in the following. The non-closure residuals in the total radiation changes in clear sky (unexplained by the kernel method) are 0.06 W m^−2^ K^−1^ in the longwave (ERA5 and H14 kernels yield the same results), and 0.07 W m^−2^ K^−1^ in the shortwave (only available from ERA5 kernels). The magnitudes of these residuals are comparable to those reported in previous studies, e.g., Huang and Huang^12^ and evidence the validity of the spectral kernel-quantified feedbacks.

**Fig. 5:**
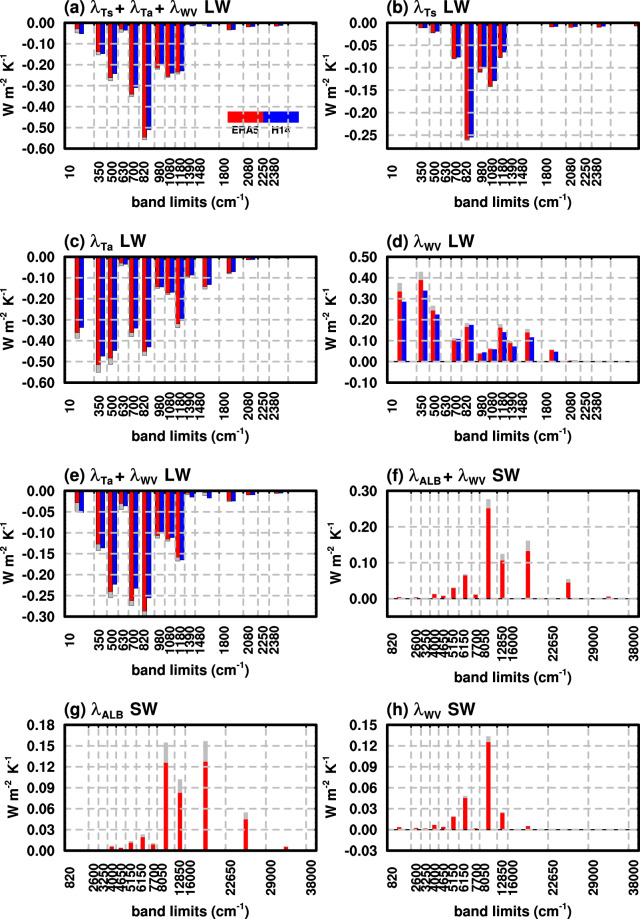
Global mean all-sky TOA spectral feedback parameters (units: W m^−2^ K^−1^) of (**a**) the total non-cloud LW feedback, and the feedbacks of (**b**) surface temperature, (**c**) air temperature, (**d**) water vapor LW, (**e**) sum of air temperature and water vapor LW feedbacks, (**f**) total non-cloud SW feedback, and the feedback of (**g**) surface albedo and (**h**) water vapor SW. Red bars represent multi-model mean diagnosed by the ERA5 kernel and blue bars that diagnosed by the H14 kernel. Grey bars represent the standard deviation of 12 models diagnosed by the ERA5 kernel.

Figure 5 shows the all-sky global mean spectral radiative feedbacks. In the longwave, the total non-cloud feedback (Fig. 5a) is mostly contributed by three spectral regions: the FIR water vapor absorption band (10–630 cm^−1^), the wings of the CO_2_ band (700–820 cm^−1^) and the mid-infrared window (820–1080 cm^−1^), with the band-integrated contributions of −0.43 W m^−2^ K^−1^, −0.34 W m^−2^ K^−1^ and −0.76 W m^−2^ K^−1^, respectively, amounting to about 72% of the total non-cloud feedback. The results based on the two different kernels (EAR5 and H14) are in good agreement. As noted in previous studies (e.g., Huang, *et al*.^15^, Jeevanjee, *et al*.^38^), there are strong compensations between air temperature and water vapor longwave feedbacks (Fig. 5c,d), which, as disclosed by the spectral kernel analysis here, is mainly in the water vapor rotational band in the FIR. However, the compensation is not exact, clearly deviating from the Simpson hypothesis^38,39^, which proposed that the effective emission of the Earth from the optically thick atmosphere tended to be fixed at a certain value with surface temperature warming, i.e., no OLR change.

In the respective air temperature and water vapor LW feedbacks (Fig. 5c,d), noticeable inter-GCM discrepancies are observed, especially in the FIR, which highlights the importance of acquiring observations in this spectral region for validating the GCMs. Figure S5 further shows the band-integrated LW feedbacks in H_2_O absorption band (10–630 cm^−1^, 1180–1800 cm^−1^), CO_2_ band (500–820 cm^−1^), window region (820–1080 cm^−1^) and O_3_ absorption band (1080–1180 cm^−1^), disclosing the model spread in these different spectral regions.

In the shortwave, both surface albedo and water vapor feedbacks are primarily contributed from the near-infrared and visible bands (Fig. 5g,h), with stronger contributions from the water vapor feedback in the near-infrared and almost equal contributions from the surface albedo and water vapor feedback in the visible band. The inter-GCM discrepancies in total non-cloud shortwave feedback (Fig. 5f) are dominated by the uncertainty in surface albedo feedback in visible bands.

### Supplementary information

Supplementary information for Band-by-band spectral radiative kernels based on the ERA5 reanalysis

## Data Availability

The ERA5 datasets can be accessed through the ECMWF website https://cds.climate.copernicus.eu/cdsapp#!/search?type=dataset. The RRTMG code can be downloaded at http://rtweb.aer.com/rrtm_frame.html. The datasets contain the multi-year averaged monthly mean spectral kernel of TOA, surface and atmosphere for surface temperature, air temperature, surface albedo and water vapor (LW and SW), as well as program scripts exemplifying their use, are available at Huang and Huang^34^.
