# Supplementary material for: Band-by-band spectral radiative kernels based on the ERA5 reanalysis
Source: Sci Data. 2024 Feb 24;11:237. doi: 10.1038/s41597-024-03080-y (PMC10894235; doi:10.1038/s41597-024-03080-y)
Supplement: Supplementary file 1 — Supplementary information for Band-by-band spectral radiative kernels based on the ERA5 reanalysis [file 41597_2024_3080_MOESM1_ESM.pdf]

Supplementary information for

Band-by-band spectral radiative kernels based on the ERA5 reanalysis

Han Huang[1], Yi Huang[1], Qiang Wei[2, 3], Yongyun Hu[3]

[1] Department of Atmospheric and Oceanic Sciences, McGill University, Montreal, Canada

[2] State Key Laboratory of Tibetan Plateau Earth System, Environment and Resources (TPESER), Institute of Tibetan Plateau Research, Chinese Academy of Sciences, Beijing, China

[3] Department of Atmospheric and Oceanic Sciences, Peking University, Beijing, China

Corresponding Authors:

Han Huang, han.huang2@mcgill.ca (ORCID: 0000-0002-9143-6453),

Yi Huang, yi.huang@mcgill.ca (ORCID: 0000-0002-5065-4198)

Figure S1. ERA5 spectral radiative kernel in clear-sky

Figure S2. Spectral feedback during ENSO diagnosed by H14 radiative kernels in clear-sky.

Figure S3. Surface energy change diagnosed by ERA5 spectral kernels during ENSO in clear-sky

Figure S4. Global mean all-sky TOA spectral feedback intensity parameters in abrupt4xCO_2 case

Figure S5. All-sky band integrated LW feedback parameters in abrupt4xCO_2 case

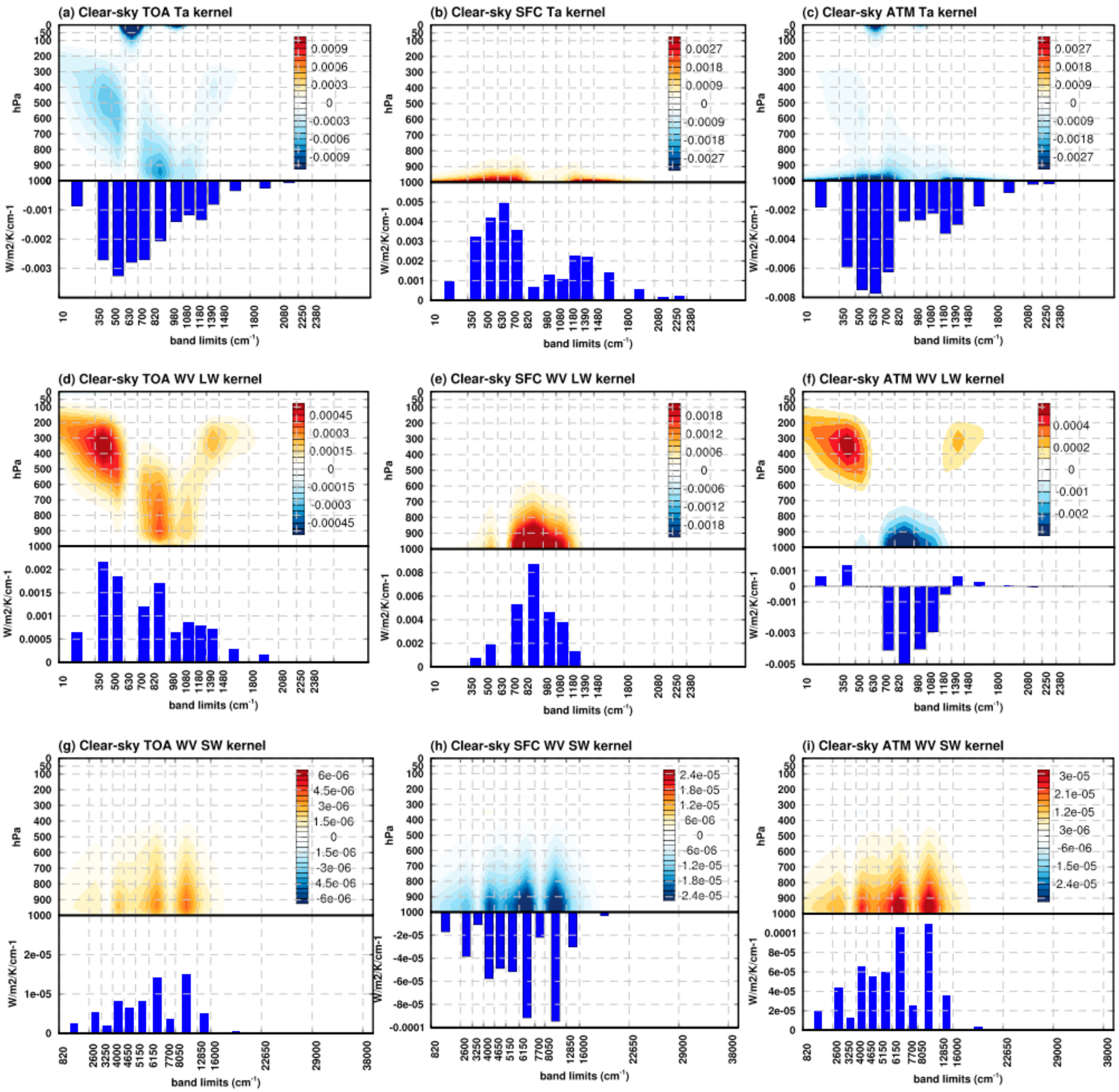

Figure S1. Similar to Figure 2 but for clear-sky spectral kernels.

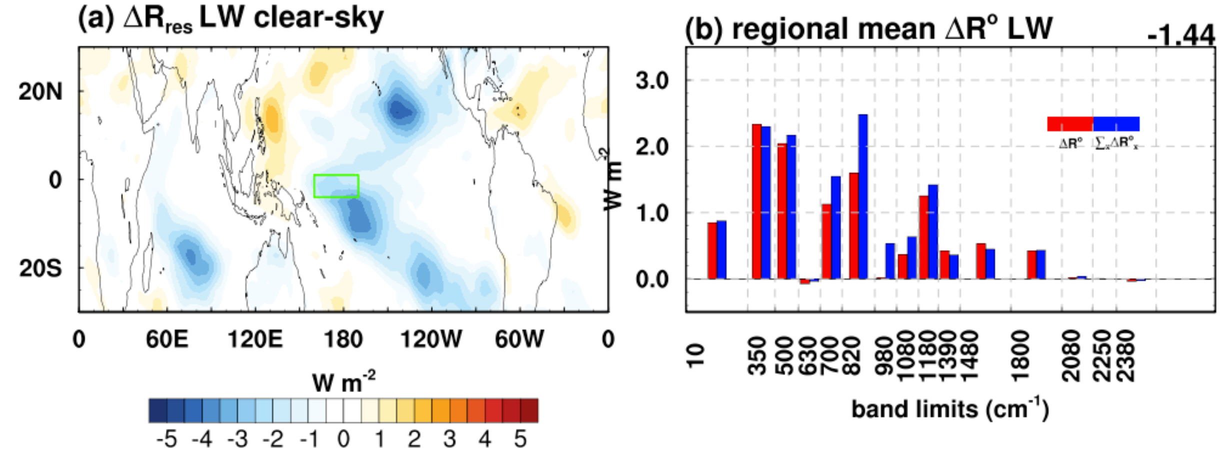

Figure S2. Clear-sky diagnosed feedbacks by H14 spectral kernels (a) for broadband residual term, (b) regional mean (as marked by the green rectangle in panel (a)) of total spectral radiative feedbacks. The number on the right corner is the regional mean residual term.

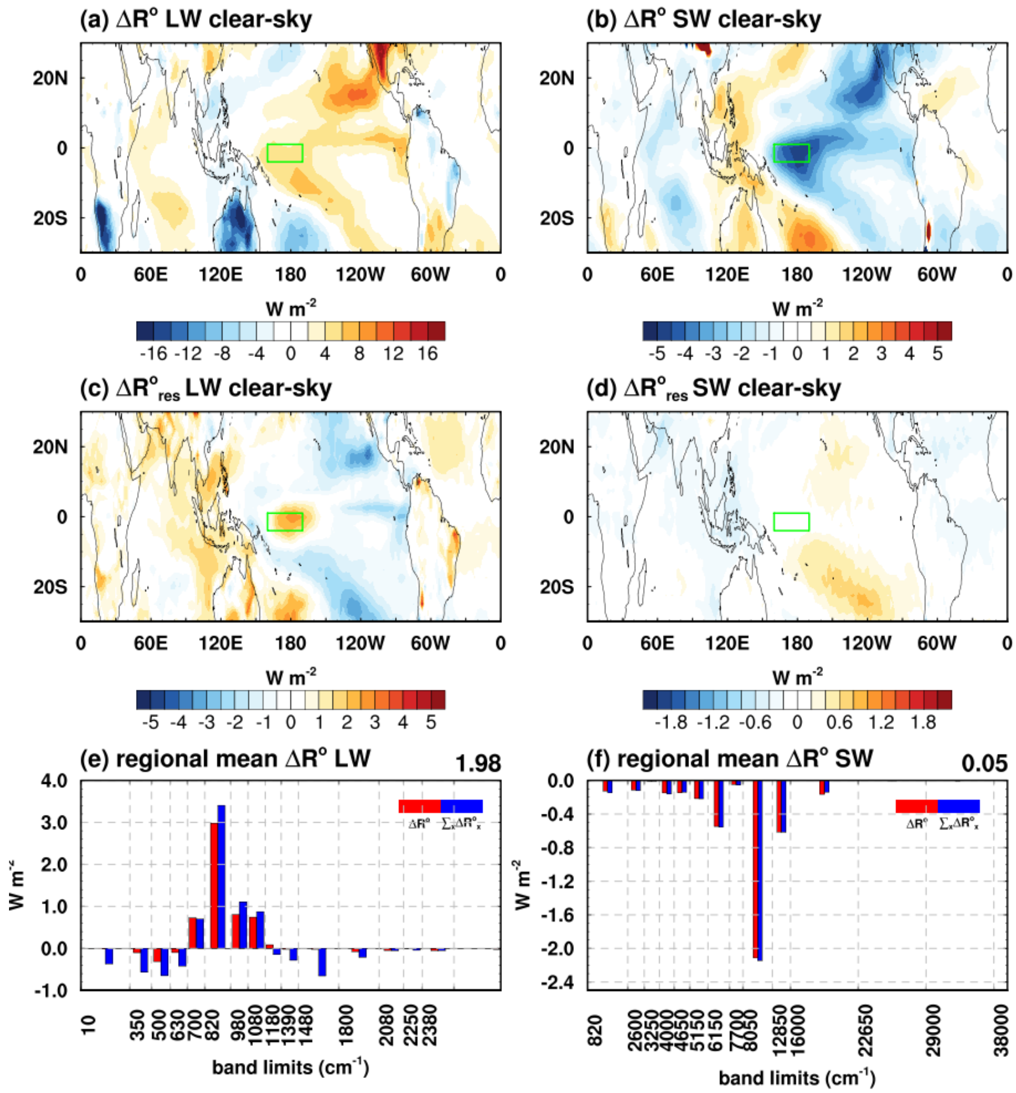

Figure S3. Similar to Figure 4, but for the surface energy budget in clear sky. (a, b) broadband radiation change simulated by RRTMG for LW and SW, (c, d) residual terms quantified by ERA5 spectral kernels for LW and SW, (e, f) regional mean (as marked by the green rectangle) spectral radiative feedbacks in each band diagnosed by ERA5 spectral radiative kernels during ENSO. The numbers on the right corner in (e, f) are the regional mean residual terms.

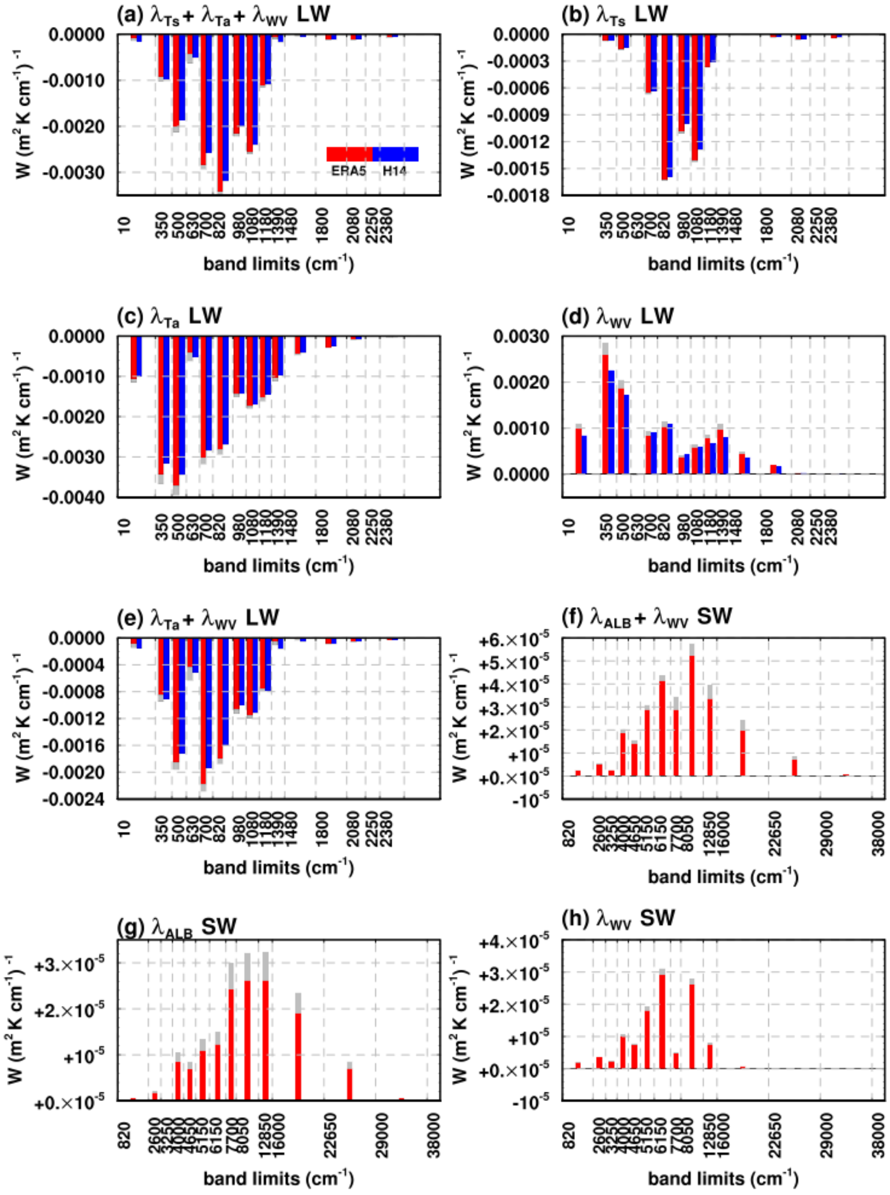

Figure S4. Similar to Figure 5, but the Global mean all-sky TOA spectral feedback intensity parameters (units: W (m^2 K cm^{-1})$^{-1}$) of (a) the total non-cloud LW feedback, and the feedbacks of (b) surface temperature, (c) air temperature, (d) water vapor LW, (e) sum of air temperature and water vapor LW feedbacks, (f) total non-cloud SW feedback, and the feedback of (g) surface albedo and (h) water vapor SW. Red bars represent multi-model mean diagnosed by the ERA5 kernel and blue bars that diagnosed by the H14 kernel. Grey bars represent the standard deviation of 12 models diagnosed by the ERA5 kernel.

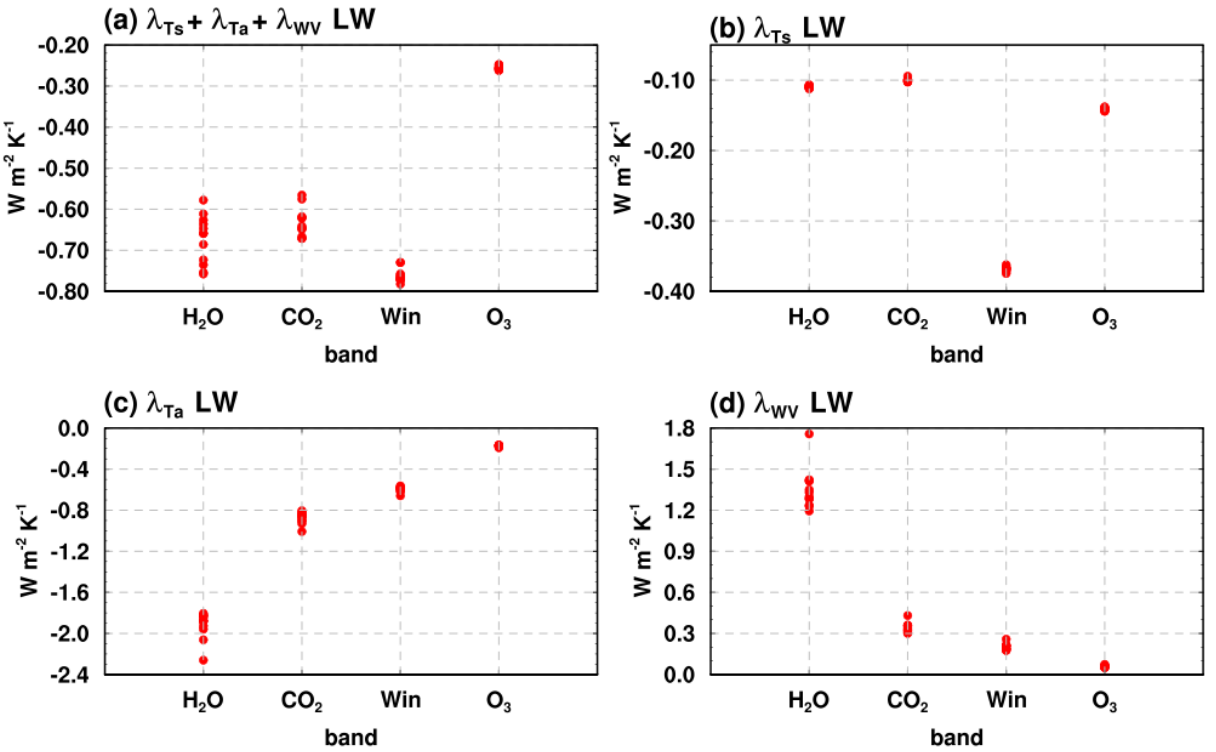

Figure S5. All-sky band integrated LW feedback parameters in abrupt4xCO_2 case for, (a) total non-cloud LW feedback, (b) surface temperature feedback, (c) air temperature feedback and (d) water vapor LW feedback, units: W m^{-2} K^{-1}. Each red dot represents one CMIP6 model. H_2O absorption band is from 10 to 630 cm^{-1} and 1180 to 1800 cm^{-1}. CO_2 band is from 500 to 820 cm^{-1}. Window region is from 820 to 1080 cm^{-1} and O_3 absorption band is from 1080 to1180 cm^{-1}.
